# GI‐530159, a novel, selective, mechanosensitive two‐pore‐domain potassium (K_2P_) channel opener, reduces rat dorsal root ganglion neuron excitability

**DOI:** 10.1111/bph.14098

**Published:** 2017-12-29

**Authors:** Alexandre J C Loucif, Pierre‐Philippe Saintot, Jia Liu, Brett M Antonio, Shannon G Zellmer, Katrina Yoger, Emma L Veale, Anna Wilbrey, Kiyoyuki Omoto, Lishuang Cao, Alex Gutteridge, Neil A Castle, Edward B Stevens, Alistair Mathie

**Affiliations:** ^1^ Pfizer NPRU Cambridge UK; ^2^ Icagen Durham NC USA; ^3^ Medway School of Pharmacy University of Kent Chatham Maritime Kent UK

## Abstract

**Background and Purpose:**

TREK two‐pore‐domain potassium (K_2P_) channels play a critical role in regulating the excitability of somatosensory nociceptive neurons and are important mediators of pain perception. An understanding of the roles of TREK channels in pain perception and, indeed, in other pathophysiological conditions, has been severely hampered by the lack of potent and/or selective activators and inhibitors. In this study, we describe a new, selective opener of TREK channels, GI‐530159.

**Experimental Approach:**

The effect of GI‐530159 on TREK channels was demonstrated using ^86^Rb efflux assays, whole‐cell and single‐channel patch‐clamp recordings from recombinant TREK channels. The expression of K_2P_2.1 (TREK1), K_2P_10.1 (TREK2) and K_2P_4.1 (TRAAK) channels was determined using transcriptome analysis from single dorsal root ganglion (DRG) cells. Current‐clamp recordings from cultured rat DRG neurons were used to measure the effect of GI‐530159 on neuronal excitability.

**Key Results:**

For recombinant human TREK1 channels, GI‐530159 had similar low EC_50_ values in Rb efflux experiments and electrophysiological recordings. It activated TREK2 channels, but it had no detectable action on TRAAK channels nor any significant effect on other K channels tested. Current‐clamp recordings from cultured rat DRG neurones showed that application of GI‐530159 at 1 μM resulted in a significant reduction in firing frequency and a small hyperpolarization of resting membrane potential.

**Conclusions and Implications:**

This study provides pharmacological evidence for the presence of mechanosensitive TREK K_2P_ channels in sensory neurones and suggests that development of selective K_2P_ channel openers like GI‐530159 could aid in the development of novel analgesic agents.

**Linked Articles:**

This article is part of a themed section on Recent Advances in Targeting Ion Channels to Treat Chronic Pain. To view the other articles in this section visit http://onlinelibrary.wiley.com/doi/10.1111/bph.v175.12/issuetoc

**Abbreviations**
BL‐1249: (5,6,7,8‐tetrahydro‐naphthalen‐1‐yl)‐[2‐(1*H*‐tetrazol‐5‐yl)‐phenyl]‐amine
CAPE: caffeic acid phenylethyl ester
CDC: cinnamyl 1,3,4‐dihydroxy‐α‐cyanocinnamate
DRG: dorsal root ganglion
GI‐530159: 4,4′‐(hexafluoroisopropylidene)bis(*p*‐phenyleneoxy)dianiline
K_2P_: two‐pore‐domain potassium

## Introduction

http://www.guidetopharmacology.org/GRAC/FamilyIntroductionForward?familyId=79 are mechanosensitive ion channels, which are members of the two‐pore‐domain (http://www.guidetopharmacology.org/GRAC/FamilyDisplayForward?familyId=79) potassium channel family (Enyedi and Czirjak, 2010; Feliciangeli *et al*., 2015; Renigunta *et al*., 2015). They contribute to background potassium conductances in many neurons and other cell types. Their activity can be regulated by a number of different physical and chemical stimuli, which include membrane stretch, membrane depolarization, heat, arachidonic acid and other polyunsaturated fatty acids (see Honoré, 2007; Noël *et al*., 2011; Mathie and Veale, 2015). There are three members of the TREK subfamily (Enyedi and Czirjak, 2010), TREK1 (http://www.guidetopharmacology.org/GRAC/ObjectDisplayForward?objectId=514&familyId=79&familyType=IC; *KCNK2*), TREK2 (http://www.guidetopharmacology.org/GRAC/ObjectDisplayForward?objectId=521&familyId=79&familyType=IC; *KCNK10*) and TRAAK (http://www.guidetopharmacology.org/GRAC/ObjectDisplayForward?objectId=516&familyId=79&familyType=IC, *KCNK4*), which share many structural and functional properties. At least in expression systems, recent evidence suggests that all three channels can form functional heterodimeric channels with each other (Blin *et al*., 2016; Lengyel *et al*., 2016; Levitz *et al*., 2016).

Increasing evidence points to an important contribution from a number of different potassium channels (see Du and Gamper, 2013; Tsantoulas and McMahon, 2014; Waxman and Zamponi, 2014), including K_2P_ channels (Alloui *et al*., 2006; Woolf and Ma, 2007; Noël *et al*., 2009; Mathie, 2010; Plant, 2012), in pain processing. Among K_2P_ channels, the strongest body of evidence from both expression functional studies highlights the importance of TREK1, TREK2 and also TRESK channels (see Mathie and Veale, 2015).

TREK1 channels are expressed in both small‐sized and medium‐sized dorsal root ganglion (DRG) neurons where they are often co‐localized with excitatory transient receptor potential cation channel subfamily V member 1 (TRPV1) channels (Maingret *et al*., 2000; Talley *et al*., 2001; Alloui *et al*., 2006; Dedman *et al*., 2009; Marsh *et al*., 2012), and enhanced expression of TREK1 is seen in DRG neurons in a mouse model of neuropathic pain (Han *et al*., 2016). TREK1 knockout mice are more sensitive than wild‐type mice to painful heat sensations near the threshold between non‐painful and painful heat (Alloui *et al*., 2006). More recently, it has been shown that the TLR7 receptor agonist, imiquimod, enhances the excitability of DRG neurons, at least in part, through blocking TREK1 channels (Lee *et al*., 2012). The pain‐relieving actions of morphine may be linked to TREK1 channel activity since morphine, acting through μ receptors, has been shown to enhance TREK1 current directly, and TREK1 knockout mice showed significantly less morphine‐induced analgesia than wild‐type animals (Devilliers *et al*., 2013). Taken together, this evidence suggests that TREK1 is a channel critical for regulating the excitability of somatosensory nociceptive neurons and, therefore, pain perception.

TREK2 channels have also been implicated in regulating somatosensory nociceptive neuron excitability (Acosta *et al*., 2014; Pereira *et al*., 2014). For example, TREK2 channels may be distinctively expressed in a subpopulation of thermosensitive neurons and regulate the perception of moderate temperature changes by altering the firing activity of certain sensory C fibres (Pereira *et al*., 2014).

A deeper understanding of the roles of TREK channels in pain perception and, indeed, in a number of other pathophysiological conditions, has been severely hampered by the lack of potent and/or selective activators and inhibitors. Up until now, the only known commercially available TREK channel activator is BL‐1249, originally identified as a putative activator of TREK1‐like currents in human bladder myocytes (Tertyshnikova *et al*., 2005). Subsequently, this compound was shown to activate TREK1 and TREK2 channels directly (Veale *et al*., 2014; Dong *et al*., 2015); however, this compound lacks the selectivity required for it to be useful, in isolation, as an indicator of TREK channel activity (see Results). Other activators of TREK channels have been described including ML67‐33 (Bagriantsev *et al*., 2013) and a number of caffeic acid esters (Danthi *et al*., 2004; Rodrigues *et al*., 2014; Vivier *et al*., 2017) (see Discussion).

In this study, we describe a new, selective opener of TREK channels, GI‐530159, and show that it reduces the excitability of small DRG neurons. A preliminary account of some of this work has been given previously (Loucif *et al*., 2015).

## Methods

### ^86^Rubidium efflux studies

CHO–hTREK1 cells were plated on 96‐well, tissue culture‐treated clear plates (92 696) the day before the assay and incubated overnight at 37°C in the presence of 1 μCi·mL^−1 86^Rubidium (Rb, NEZ072, Perkin Elmer, Waltham MA, USA). Prior to commencement of the assay, the ^86^Rb spiked media were removed from the plate, and cells were washed four times with 100 μL of a 5 mM K^+^ ‘Earle's balance salt solution’ (EBSS) buffer containing 135 mM NaCl, 5.4 mM KCl, 1.8 mM CaCl_2_, 0.8 mM MgCl_2_,10 mM HEPES and 5 mM glucose, pH 7.4 (adjusted with NaOH). After the last wash, the buffer was removed, replaced with 100 μL of either 5 mM K^+^ EBSS alone or containing different concentrations of GI‐530159 or 70 mM K^+^ EBSS [containing 70 mM NaCl, 70 mM KCl, 1.8 mM CaCl_2_, 0.8 mM MgCl_2_, 10 mM HEPES and 5 mM glucose, pH 7.4 (adjusted with NaOH)], alone or containing reference inhibitor 100 μM CP‐338818 (C2499, Sigma‐Aldrich, St Louis MO, USA). After plate was incubated for 60 min at room assay, buffer was removed from the plates and transferred to a counting plate. The cells were then lysed with 100 μL of 0.1% SDS, and the lysate transferred to a second counting plate. Both counting plates were then read on the Wallac Microbeta to measure ^86^Rb content [counts per min (CPM)]. % ^86^Rb efflux values were calculated as follows:

$${}^{86}\mathrm{Rb}\;\text{efflux}\;\left(\%\text{cell content}\right)=\left(\left(\mathrm{CPM}\;\text{in efflux buffer}\right)/\left(\mathrm{CPM}\;\text{in efflux buffer}+\mathrm{CPM}\;\text{in cell lysate}\right)\right)*100.$$

Magnitude of ^86^Rb efflux stimulation was calculated as follows:

$$\%\text{Activation}=\left(\left(\%\text{efflux test well}-\text{efflux in}\;5.4\;\mathrm{mM}\;K^{+}\;\text{EBSS}\right)/\left(\text{Efflux in}\;70\;\mathrm{mM}\;K^{+}\;\text{EBSS}-\text{efflux in}\;5.4\;\mathrm{mM}\;K^{+}\;\text{EBSS}\right)\right)*100.$$

Half maximal activation of ^86^Rb efflux (EC_50_) value was calculated using IDBS Xlfit using a four‐parameter logistic equation and expressed as mean ± SEM.

### Electrophysiological recordings

Electrophysiological studies were performed on CHO or HEK293 cells, stably expressing human recombinant TREK1; tsA‐201 cells transiently transfected with human recombinant K2P channels and acutely dissociated rat DRG cells, all at room temperature (22–24°C).

### Automated whole‐cell voltage clamp

Automated patch‐clamp studies were performed with the Qpatch Sophion Bioscience A/S (Ballerup, Denmark) automated patch‐clamp platform. Sixteen‐well Qplates were loaded with CHO cells stably expression human recombinant TREK1 (CHO–TREK1 cells) in external buffer containing (in mM) 148 NaCl, 5 KCl, 1 CaCl_2_, 1 MgCl_2_, 10 HEPES and 10 glucose. Internal recording solution was (in mM) 100 K gluconate, 40 KCl, 1 MgCl_2_, 10 BAPTA, 10 HEPES and 1 MgATP.

### Whole‐cell recording from HEK293 cells stably expressing TREK1

Studies were performed using a conventional patch clamp and Multiclamp 700B and pCLAMP software (Molecular Devices, Sunnyvale, CA, USA). For conventional manual patch clamp, glass coverslips containing HEK293 cells were perfused with an extracellular solution (ECS) containing (in mM): 135 NaCl, 4.7 KCl, 1 CaCl_2_, 1 MgCl_2_, 10 HEPES and 10 glucose, pH 7.4, with NaOH (310 mOsmol·L^−1^). The patch pipette was filled with (in mM): 130 KCl, 1 MgCl_2_, 5 MgATP, 10 HEPES and 10 BAPTA, pH adjusted to 7.3 with KOH (290 mOsmol·L^−1^).

### Single‐channel recordings

Human TREK1 single channels were recorded in excised inside‐out membrane patches using an Axopatch 200B patch‐clamp amplifier and pCLAMP 10 software. Inside‐out membrane patches were excised from HEK293 cells expressing human TREK1 channels. Sylguard® 184‐coated glass pipettes with resistance around 10–15 MΩ were used. Pipette and bath compositions for inside‐out patches were opposite to those used in voltage‐clamp, that is, the pipette solution on the extracellular face of the channels contained 130 mM K and the bath solution on the intracellular face of the channel contained 4.7 mM K. Channel activities were recorded at +60 mV. The action of a hTREK1 opener was tested by addition to the intracellular solution. Single‐channel records were filtered with an eight‐pole Bessel filter (model LHBF‐48X, npi electronic GmbH, Tamm, Germany) at a corner frequency (*fc*) of 2 kHz and acquired using a DigiData 1440A interface and pCLAMP 10 software at a sampling rate of 20 kHz. Single‐channel traces were generated without further filtering.

### Whole‐cell recording from tsA‐201 cells transiently expressing TREK1 and other K_2P_ channels

Recordings of hTREK1, hTREK1∆N, hTREK2, hTRAAK and hhttp://www.guidetopharmacology.org/GRAC/ObjectDisplayForward?objectId=523 currents from transiently transfected tsA‐201 cells were performed using methods as described in Veale and Mathie (2016). Briefly, the pcDNA3.1 vector was cloned with the gene of interest, and these vectors and a similar vector containing GFP were incorporated into the tsA201 cells (0.5 μg per well) using the calcium phosphate method. The cells were incubated for 6–12 h at 37°C in 95% oxygen and 5% carbon dioxide. Cells were then washed using a PBS solution and used for experiments after 24 h.

Currents were recorded using whole‐cell patch‐clamp using an Axopatch 1D amplifier. Cells were placed in a recording chamber filled with an external medium composed of 145 mM, NaCl, 2.5 mM KCl, 3 mM MgCl_2_, 1 mM CaCl_2_ and 10 mM HEPES (pH to 7.4, using NaOH). The internal medium used in the glass pipette comprised 150 mM KCl, 3 mM MgCl_2_, 5 mM (or 0.1 mM) EGTA and 10 mM HEPES (pH to 7.4, using KOH). GI‐530159 was applied by bath perfusion. Currents were recorded and analysed using pCLAMP 10.2 software Microsoft Excel. The voltage protocol used for recording current through K_2P_ channels was as previously described (Veale and Mathie, 2016). For analysis, we measured the current difference between the −80 and −40 mV steps.

### Recordings from dorsal root ganglion neurons

Acutely dissociated rat DRG cells were obtained according to a previously described protocol (Passmore, 2005). All animal care and experimental procedures complied with guidelines and were approved by the Pfizer Neusentis Institutional Animal Use and Care Committee. Animal studies are reported in compliance with the ARRIVE guidelines (Kilkenny *et al*., 2010; McGrath and Lilley, 2015).Cells were plated on coated coverslips (poly‐d‐lysine/laminin, BD Biosciences, San Jose, CA, USA) prior to patching on the same day. Cells were perfused with an extracellular solution containing (in mM): 135 NaCl, 4.7 KCl, 1 CaCl_2_, 1 MgCl_2_, 10 HEPES and 10 glucose, pH 7.4, with NaOH (310 mOsmol·L^−1^). Pipette solution contained (in mM): 130 KCl, 1 MgCl_2_, 5 MgATP, 10 HEPES and 5 EGTA, pH adjusted to 7.3 with KOH (290 mOsmol·L^−1^). For whole‐cell recordings, currents were filtered at 2 kHz and sampled at 5 kHz. Series resistance was compensated for by up to 80%. For current‐clamp recordings, action potential firing and resting membrane potential (RMP) were recorded from small cells (<30 pA). GI‐530159 was dissolved in 0.3% DMSO as 10 mM stock and applied in the vicinity of cells using a gravity fed perfusion system (MSC‐200, Bio‐Logic SAS, Claix, France).

Data were analysed using pCLAMP or Qpatch software or Spike2 (Cambridge Electronic Device, Cambridge, UK) and Prism 6.0 (GraphPad, San Diego, CA, USA) software.

### Transcriptome analysis from single DRG cells

Lumbar DRGs from four rats were dissociated then passed through a BSA gradient. Individual cells were loaded onto Fluidigm C1 17–25 μm size chips for single‐cell preparation. Three replicates were performed. Cell collection chambers were viewed down the microscope to exclude any that had multiple cells/chunks of debris. The C1 lysed, reverse transcribed and preamplified the cells, using the SMARTseq protocol. Sequencing libraries were prepared and multiplexed 96 ways using the Nextera XT kit (Illumina, San Diego CA, USA). Libraries were quantified using the Qubit High Sensitivity DNA assay (Thermo Fisher Scientific, Waltham MA, USA) and library quantification kits (KAPA Biosystems, Wilmington MA, USA) and pooled in equal amounts for single‐end sequencing on an Illumina Nextseq 500. Sequencing data were aligned, and gene level expression measured using spliced transcripts alignment and reconstruction tool (Dobin *et al*., 2013) and assigned to genomic features using featureCounts (Liao *et al*., 2014) and Ensembl gene annotations. Gene read counts were converted to fragments per kilobase per million (FPKM) and log_2_ transformed. In total, ~180 cells were successfully processed of which 120 were defined as neurons, based on marker expression.

### Group sizes

The exact group size (*n*) for each experimental group/condition is provided, and ‘*n*’ refers to independent values, not replicates. Data subjected to statistical analysis have *n* of at least five per group.

### Randomization

When comparisons are made between different recording conditions or different, mutated, forms of a channel, recordings were alternated between one condition and the other on a given day.

### Blinding

No blinding was undertaken in this study. It is not a usual procedure for this form of study and cannot be applied retrospectively.

### Normalization

Normalization of responses was carried out in some experiments (Rb flux experiments and some electrophysiological experiments) to allow comparison with standardized responses and to minimize the influence of variable baseline levels of current activity on comparisons of percentage enhancements between one experimental platform and another.

### Statistical comparison

Group mean values and statistical analysis used independent values. When comparing groups, a level of probability (*P* < 0.05) was deemed to constitute the threshold for statistical significance. For statistical comparisons of currents in the absence and presence of GI‐530159 (1 μM) (Figures 3 and 5), each *n* value represents a recording from a cell on an independent coverslip on different recording days. Comparisons were made using two‐tailed paired *t*‐tests. To compare the degree of enhancement by GI‐530159 (1 μM) between TREK1, TREK2 and TREK1∆N channels (Figure 5D), one‐way ANOVA, followed by a Dunnett's multiple comparisons test was used. In Figure 7D, E, we compared the membrane potential before and after addition of GI‐530159 (1 μM) in 14 neurons from seven different DRG preparations (seven different animals). Comparisons were made using two‐tailed paired *t*‐tests both for each of the 14 neurons (*n* = 14, Figure 7D) and for the averaged response in neurons from a single animal to give *n* = 1 for each animal used (so *n* = 7 animals, Figure 7E).

Statistical analyses were carried out using Graphpad Prism 6.0 (Graphpad). The data and statistical analysis comply with the recommendations on experimental design and analysis in pharmacology (Curtis *et al*., 2015).

### Nomenclature of targets and ligands

Key protein targets and ligands in this article are hyperlinked to corresponding entries in http://www.guidetopharmacology.org, the common portal for data from the IUPHAR/BPS Guide to PHARMACOLOGY (Southan *et al*., 2016), and are permanently archived in the Concise Guide to PHARMACOLOGY 2017/18 (Alexander *et al*., 2017a,b).

## Results

### Activation of recombinant TREK channels by GI‐530139

**Figure 1:**
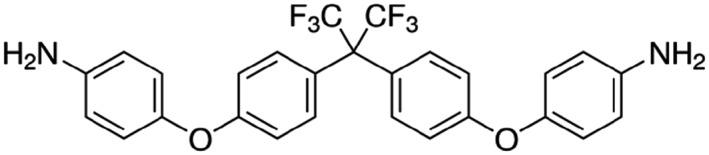
Structure of GI‐530159.

The structure of GI‐530139 (originally ICA‐069771) is shown in Figure 1. The compound has not previously been shown to have an effect on any identified biological target, but it is available commercially (Sigma‐Aldrich) as a primary amide for generating non‐stick coatings.

**Figure 2:**
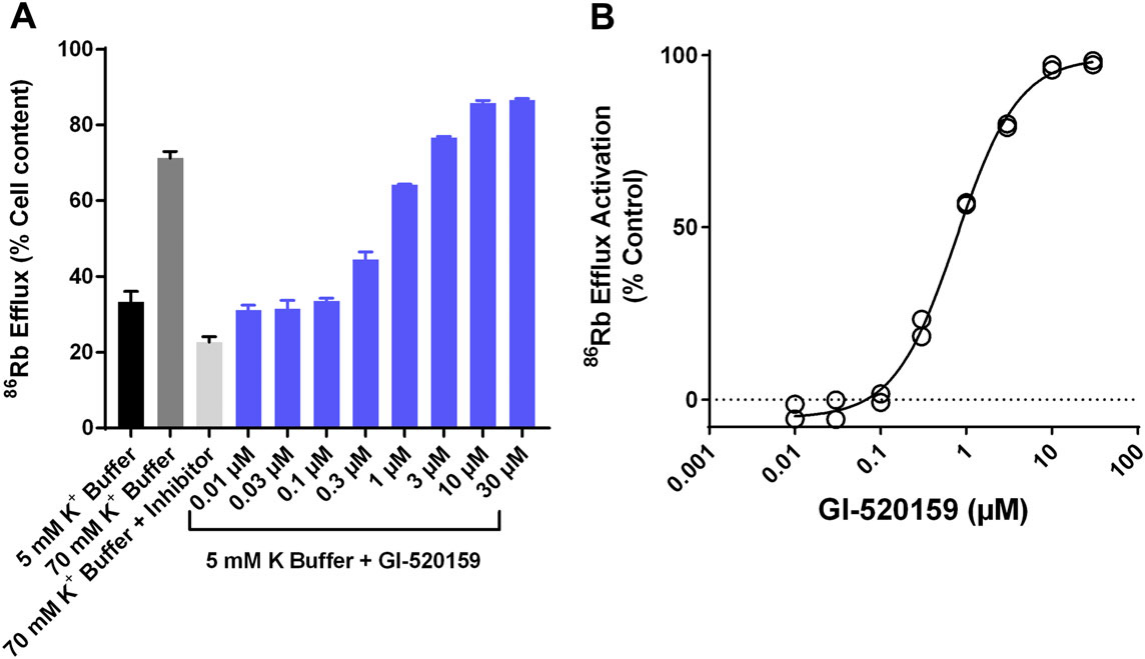
Effect of GI‐530159 on ^86^Rb flux through TREK1 channels. (A) Exemplar values of ^86^Rb efflux from 96‐well plate containing monolayers of CHO–hTREK1 during 60 min exposure to normal 5 mM K EBSS buffer, 70 mM K buffer, 70 mM KCl buffer and reference inhibitor (100 μM CP‐338818) as well increasing concentrations of GI‐530159 made up in 5 mM K EBSS buffer. ^86^Rb efflux is expressed as % of cell content at beginning of experiment (*n* = 3 for each concentration of GI‐530159, *n* = 24 for 5 mM K buffer). (B) Concentration‐dependence of stimulation of ^86^Rb efflux by GI‐530159 normalized to the response observed with 70 mM K EBSS buffer. Maximal flux (142 ± 2%) and EC_50_ (0.76 ± 0.1 μM) were derived from fit of logistic equation to data.

GI‐530159 was originally identified as a putative TREK1 channel activator using an ^86^Rb screen of a CHO–hTREK1 cell line (Figure 2). Figure 2A shows exemplar values for ^86^Rb efflux from CHO–hTREK1 cells after a 60 min exposure to either normal (5 mM K) external solution, a high potassium (70 mM K) solution in the absence and presence of a reference inhibitor (100 μM CP‐338818) or the presence of increasing concentrations of GI‐530159 in normal external solution. ^86^Rb efflux is expressed as % of total cell content at beginning of experiment. The concentration–response relationship for ^86^Rb efflux by GI‐530159 (normalized to the response observed with 70 mM K) is shown in Figure 2B. From a fit of the logistic equation to the data, the maximum effect of GI‐530159 was 142 ± 2% of that seen with 70 mM K, and the EC_50_ for the compound was 0.76 ± 0.1 μM.

**Figure 3:**
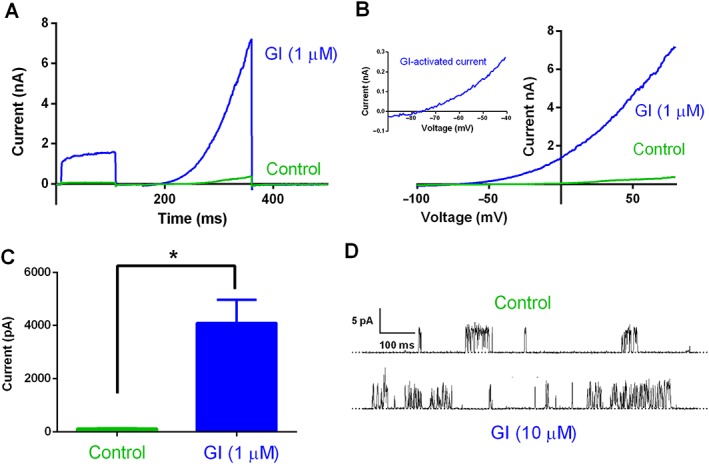
Effect of GI‐530159 on stably transfected TREK1 channel currents. (A) Whole‐cell current recordings of TREK1 channels stably transfected in HEK293 cells, in the presence (blue) and absence (green) of GI‐530159 (1 μM). (B) Current–voltage relationship for TREK1 currents in the presence (blue) and absence of GI‐530159. The inset shows the current–voltage relationship for GI‐530159‐activated current. (C) Control current, measured at 0 mV (green), was significantly enhanced by 1 μM GI‐530139 (blue, *n* = 6, *P < 0.05, paired *t*‐test). Each *n* value represents a recording from a cell on an independent coverslip on different recording days. (D) Representative single‐channel records of hTREK‐1 in excised inside‐out membrane patches (12 inside‐out patch recordings in total) from HEK293 cells in the presence and absence of GI‐530159 (10 μM). Dotted line indicates the closed channel state, and upward deflections correspond to channel openings. Membrane patches were voltage clamped at +60 mV at room temperature.

Figure 3 shows the effect of GI‐530139 on hTREK1 channel currents in HEK293 cells recorded using conventional manual patch clamp. The enhancement of current is illustrated for 1 μM GI‐530139 (Figure 3A) and can be seen both in the voltage step from −80 to 0 mV and in the voltage ramp from −100 to +80 mV (Figure 3B). The current–voltage relationship for GI‐530139 enhanced current was obtained by subtracting the current in the absence of the compound (but in the presence of the compound vehicle, DMSO) from that obtained in its presence. This is shown as an inset to Figure 3B and illustrates that the TREK1 current is enhanced at all voltages by GI‐530139 and that the enhanced current is outwardly rectifying. The GI‐530139‐enhanced current reverses close to −80 mV, and there is clear increased current at voltages between −70 and −40 mV by GI‐530139, voltages that encompass the normal resting membrane potential of small DRG neurons (see Figure 7, below). Current, measured at 0 mV, was significantly increased from 116 ± 15 pA in the presence of DMSO to 4090 ± 878 pA following addition of 1 μM GI‐530139 (*n* = 6, *P* < 0.05, paired *t*‐test, Figure 3C). The same concentration of GI‐530139 is also effective at reversibly enhancing current through hTREK1 channels in stably transfected CHO cells, recorded using the Qpatch automated patch‐clamp system (Supporting Information Figure S1) and hTREK1 channels transiently expressed in tsA‐201 cells (Figure 5A), although for these two latter platforms, the percentage enhancement is lower, at least in part because basal current activity is higher (see Discussion).

The activity of single hTREK1 channels measured at +60 mV in the inside‐out manual patch configuration are enhanced by GI‐530139 (Figure 3D). From the traces, it can be seen that the single‐channel current amplitude was not altered by application of GI‐530139 (10 μM) to the intracellular side of the membrane, rather the channel open probability was increased. In the recording from which these exemplar traces were taken, the open probability increased from 0.009 to 0.025 following application of GI‐530139. Enhancement of channel open probability by GI‐53019 was observed in 12 inside‐out patch recordings, three of which contained just a single active TREK‐1 channel. This provides strong evidence that GI‐530139 acts directly on TREK1 channels to enhance current rather than acting indirectly through a second messenger pathway. This is similar to the direct action on TREK channels shown previously for BL‐1249 (Dong *et al*., 2015).

**Figure 4:**
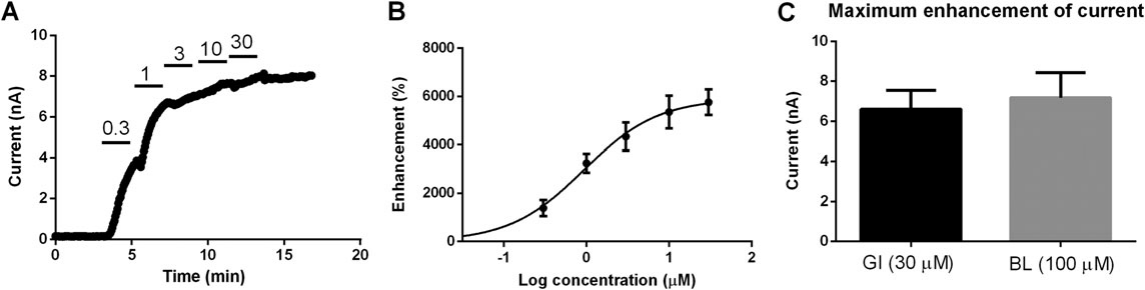
Concentration–response curve for GI‐530159 on stably transfected TREK1 channels. (A) Representative example of cumulative concentration–response curve for GI‐530159 on TREK1 current stably transfected in HEK293 cells. Current was measured at 0 mV. (B) Concentration–response curve reveals EC_50_ of 0.9 μM for GI‐530159 (*n* = 6 for each concentration). Each *n* value represents a recording from a cell on an independent coverslip on different recording days. (C) Maximum current enhancement by GI‐530159 (*n* = 6) is similar to that seen for BL‐1249 (*n* = 7) for TREK1 currents in stably transfected HEK293 cells.

Concentration–response data were obtained for GI‐530139 over a range of concentrations from 0.1 to 30 μM, measured at 0 mV. These data show that GI‐530139 at 10–30 μM can maximally activate the channel (Figure 4A, B) with a GI‐530139 enhanced current of 6.6 ± 0.9 nA (*n* = 5) at 30 μM. This can be compared with a maximal enhanced current by BL‐1249 at 100 μM of 7.2 ± 1.3 nA (*n* = 7) (Figure 4C). A fit of the concentration–response curve for GI‐530139 gives an EC_50_ of 0.89 ± 0.3 μM, very close to that seen for the Rb efflux data above (0.76 μM, Figure 2B). By comparison, BL‐1249 has an EC_50_ of around 1 μM in electrophysiological experiments (Cao *et al*., 2010). Thus, the extent of TREK1 channel activation is similar between GI‐530139 and BL‐1249, and both compounds show similar potency.

The compounds do, however, show important differences in their selectivity as shown by studies using both manual patch‐clamp and flux assay platforms. Within K channel families, BL‐1249 has a significant effect on other ion channels, which is not seen with GI‐530139. For example, BL‐1249 produces significant inhibition of BK (K_Ca_1.1), K_v_7.2 (KCNQ2), K_v_7.3(KCNQ3) and Na_V_1.7 channels at a concentration of 10 μM. By contrast, using manual patch clamp in recombinant cell lines, the following effects were observed against other potassium channels with GI‐530139 at 10 μM: K_2P_18.1 (TRESK; −2.0 ± 3.5%, *n* = 4), K_Ca_1.1 (BK; −3.3 ± 6.7%, *n* = 4) and K_v_11.1 (hERG: −7.3 ± 5.1%, *n* = 5). Furthermore, GI‐530139 had no activator effects on other potassium channels TASK2 (K_2P_5.1), K_v_7.1, K_v_7.2 and K_v_7.3 using Rb^+^ efflux assays or against sodium channels Na_V_1.2, Na_V_1.7 and Na_V_1.8 (EC_50_ >100 μM).

In addition to activating TREK1 channels, BL‐1249 is a strong activator of TASK3 (K_2P_9.1) channels (see Cao *et al*., 2010). By contrast, GI‐530139 had negligible effect on TASK3 channels at a concentration (1 μM) that is around 50% effective at activating TREK1 channels, with an activated current of 0.02 ± 0.005 nA (*n* = 5) for TASK3 channels compared with 4.0 ± 0.9 nA (*n* = 6) for TREK1 channels.

**Figure 5:**
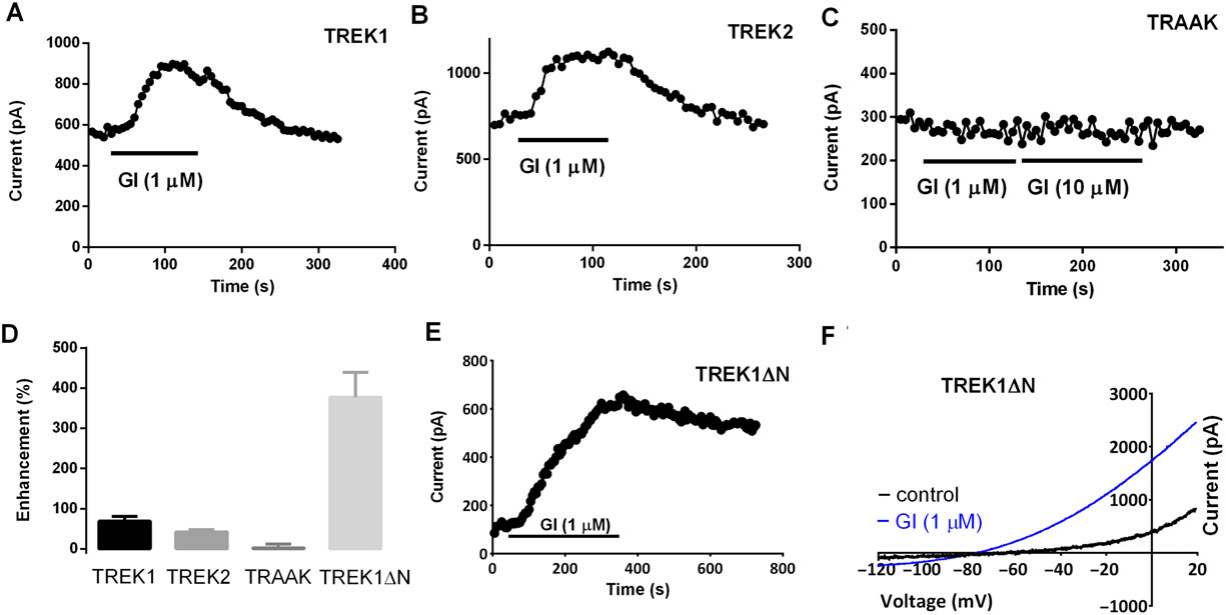
Effect of GI‐530159 on TREK1, TREK2, TRAAK and TREK1ΔN channels transiently transfected in tsA‐201 cells. (A, B) GI‐530159 activates TREK1 and TREK2 channels transiently transfected in tsA‐201 cells. (C) GI‐530159 has no detectable activation of TRAAK channels. (D) Effect of GI‐530159 on TREK1 (*n* = 6), TREK2 (*n* = 6), TRAAK (*n* = 7) and TREK1ΔN (*n* = 5) channels in transiently transfected tsA‐201 cells. (E, F) GI‐530159 activates TREK1ΔN channels. Each *n* value represents a recording from a cell on an independent coverslip on different recording days. The degree of enhancement of current through TREK1 channels was found to be not significantly different from that through TREK2 channels but was significantly smaller than that through TREK1ΔN channels (one‐way ANOVA, followed by Dunnett's multiple comparisons test; *P*<0.05).

However, within the TREK family, GI‐530139 is not completely selective for TREK1 channels. Figure 5A, D shows activation of TREK1 channels transiently transfected into tsA‐201 cells by GI‐530139 (1 μM, 69 ± 12%, *n* = 6). The percentage enhancement by GI‐530139 in these cells is much smaller than that seen in stably transfected HEK293 cells but comparable with that seen in automated patch recordings for stably transfected CHO cells (Supporting Information Figure S1), primarily because of differences in basal current level. The enhancement of TREK2 channels by 1 μM GI‐530139 in transiently transfected tsA‐201 cells was 42 ± 6%, *n* = 6 (Figure 5B, D). TRAAK channels are not activated by 1 μM GI‐530139 (Figure 5C, D, 2 ± 10%, *n* = 7), and there is no detectable effect on TRAAK channels even at a concentration of 10 μM (see Figure 5C), in contrast to the clear enhancement of TRAAK channels seen with BL‐1249 (Supporting Information Figure S2). GI‐530139 produces activation of the short form of TREK1 channels (TREK1ΔN), formed by alternative translation initiation (Thomas *et al*., 2008; Veale *et al*., 2010; Veale *et al*., 2014) (Figure 5D–F). The enhancement of TREK1ΔN channels was 377 ± 57% (*n* = 5). The degree of enhancement of current through TREK1 channels was found to be not significantly different from that through TREK2 channels but was significantly smaller than that through TREK1∆N channels (one‐way ANOVA, followed by Dunnett's multiple comparisons test, significance at the 0.05 level). It is not clear to what degree this differential effect between TREK1 and TREK1∆N is due to the much smaller background activity of TREK1∆N channels. We observed no effect of GI‐530159 on THIK1 (K_2P_13.1) channels (1 μM, 0 ± 3%, *n* = 7).

### TREK channel expression in DRG neurons

There are two major classes of nociceptive somatosensory neuron, the A fibres, which are lightly myelinated and have intermediate to large cell body sizes, and C fibres, which are unmyelinated and have smaller cell bodies (Tsantoulas and McMahon, 2014). C fibres can be subdivided, based on the expression of neuropeptides such as substance P, binding to the plant lectin IB4, or expression of thermosensitive TRP channels (Tsunozaki and Bautista, 2009). Acute localized pain is primarily transmitted by Aδ fibres, whilst diffuse pain, including itch, is mediated by C fibres (Plant, 2012).

Previous work suggests that TREK1 channels are expressed in both small‐sized and medium‐sized DRG neurons where they are often co‐localized with excitatory TRPV1 channels (see Introduction), whilst TREK2 channels have been shown to be selectively expressed in IB4 binding (non‐peptidergic) C nociceptors (Acosta *et al*., 2014).

**Figure 6:**
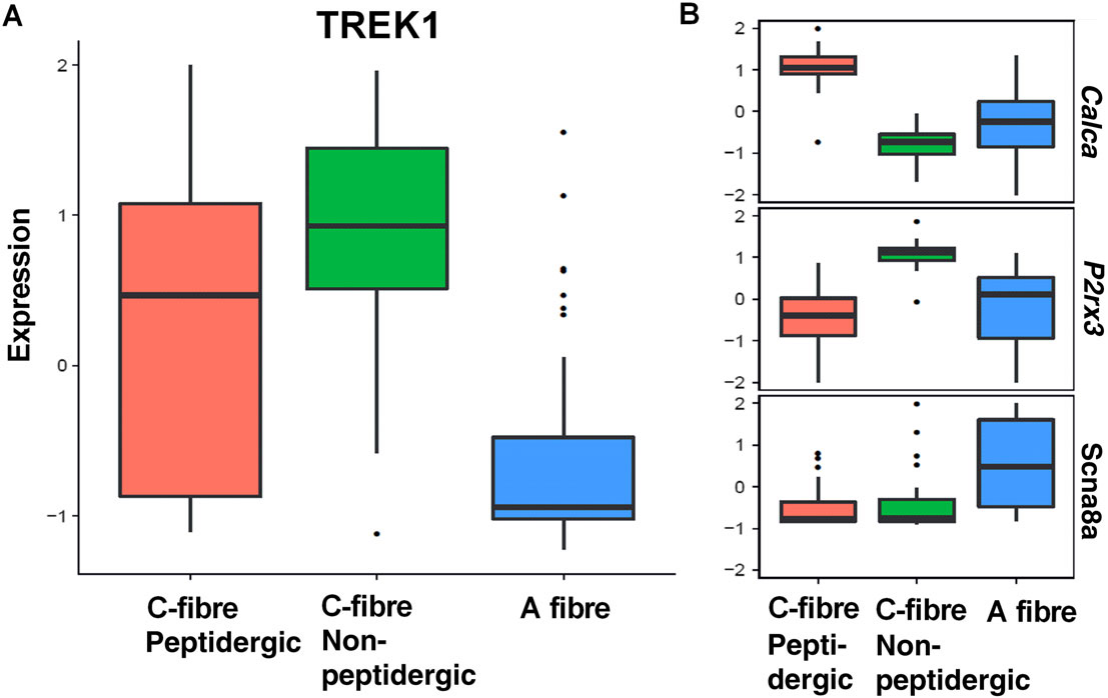
Single DRG neuron transcriptome – TREK1 channels. (A) Differential TREK1 expression in single peptidergic C fibres, non‐peptidergic C fibres and A fibres. (B) Comparative expression of selective markers for peptidergic C fibres (*Calca*), non‐peptidergic C fibres (*P2rx3*) and A fibres (*Scn8a*). Expression is given as log_2_ FPKM. Data are from 120 individual DRG neurons isolated from four rats.

Single‐cell RNA sequencing was used to characterize TREK1, TREK2 and TRAAK expression levels in individual rat DRG neurons. Of 180 cells processed, 120 were classified as neuronal based on marker expression. These neurons were then clustered into three distinct subtypes (peptidergic C fibre, non‐peptidergic C fibre or A fibre) defined by the expression of a variety of marker genes. The panels on the right‐hand side of Figure 6B and Supporting Information Figures S3 and S4 show the expression of three key markers [http://www.guidetopharmacology.org/GRAC/LigandDisplayForward?ligandId=3586, http://www.guidetopharmacology.org/GRAC/ObjectDisplayForward?objectId=480&familyId=77&familyType=IC, http://www.guidetopharmacology.org/GRAC/ObjectDisplayForward?objectId=583] across all 120 cells divided into those three groups. The *y*‐axis is a normalized measure of gene expression (log‐transformed FPKM), and the three boxes indicate the median expression of the gene within a given cluster and the 25th–75th quantiles of the expression. The whiskers and individual points show the expression in ‘outlier’ cells. For the indicated markers, *Calca* expression is the highest in peptidergic C fibres, *P2rx3* is the highest in non‐peptidergic, small diameter C fibres and *Scn8a* is the highest in A fibres.

For the TREK K_2P_ family members, TREK1 is expressed in non‐peptidergic C fibres, expression within peptidergic C fibres is variable, whilst expression in A fibres is low (Figure 6A). For TREK2, there is low expression in non‐peptidergic C fibres but greater expression in A fibres (although variably) and variable expression within peptidergic C fibres (Supporting Information Figure S3). Expression of TRAAK is quite variable within each subtype (Supporting Information Figure S4).

### Reduction in DRG neuron excitability by GI‐530139

**Figure 7:**
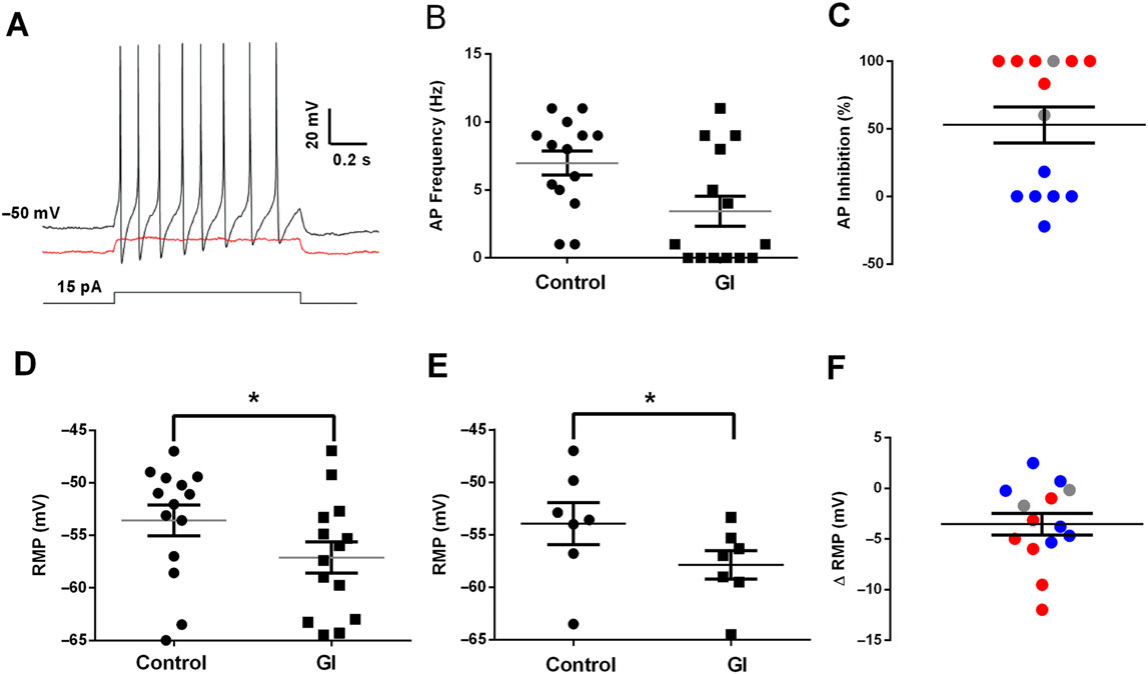
Effect of GI‐530159 on small DRG neuron firing properties. (A) Normal firing of individual DRG neuron in response to current injection (black trace) is inhibited by GI‐530159 (1 μM, red trace). (B, C) GI‐530159 inhibits action potential firing in some small DRG neurons. (D–F) GI‐530159 (1 μM) hyperpolarizes the membrane potential of small DRG neurons. In (D), membrane potential in each individual neuron is hyperpolarized from −53.6 ± 1.5 to −57.1 ± 1.5 mV (*n* = 14 individual neurons, *P* < 0.05, paired *t*‐test) by GI‐530159 (1 μM). In (E), the average membrane potential in neurons from a given rat is significantly hyperpolarized from −54.0 ± 2.0 to −57.8 ± 1.4 mV (*n* = 7 rats, *P* < 0.05, paired *t*‐test, Figure 7D) by GI‐530159 (1 μM). In (C, F), six neurons with clear inhibition of firing are shown in red, and six neurons where there was no inhibition of firing are in blue. Two neurons, which were not clearly defined, are shown in grey. Each recorded neuron was from an independent coverslip, and the 14 neurons were taken from seven independent preparations of DRG neurons.

Using current‐clamp recordings from small rat DRG neurones in short‐term culture, application of GI‐530139 at 1 μM (close to the EC_50_ for GI‐530139 on TREK1 channels) resulted in a significant hyperpolarization of the RMP of these neurons from −53.6 ± 1.5 to −57.1 ± 1.5 mV (*n* = 14 individual neurons, *P* < 0.05, paired *t*‐test, Figure 7A, D, F) and from −54.0 ± 2.0 to −57.8 ± 1.4 mV (*n* = 7 rats, *P* < 0.05, paired *t*‐test, Figure 7E). An exemplar DRG neuron is illustrated in Figure 7A, which fired repeated action potentials following a 15 pA depolarizing current injection. Following application of GI‐530139 (1 μM), however, the membrane potential was hyperpolarized, and 15 pA of depolarizing current injection no longer evoked action potential firing. Overall, application of GI‐530139 led to a reduction in cell firing frequency in response to depolarizing current injection (15 pA). However, the effects on cell firing were variable from cell to cell (Figure 7B, C). Of the 14 cells tested, six showed a complete abolition in firing frequency in the presence of GI‐530139 whilst two showed a reduction. The firing of six neurons (43%), however, was unaltered following application of GI‐530139 (Figure 7C). The six cells with no change in firing are shown in blue in Figure 7C, F, whilst the six cells with abolition of firing are shown in red in Figure 7C, F.

A similar effect on DRG neuron firing was observed with BL‐1249 (Cao *et al*., 2010). However, these data are more difficult to interpret because of the lack of selectivity for TREK channels seen with BL‐1249. Indeed, in voltage‐clamp recordings from DRG neurons, a clear block of Na current was seen with BL‐1249 (an effect not observed with GI‐530139), which would contribute to effects on firing and compromise interpretation of results with this compound.

## Discussion

In this study, we describe a novel activator of TREK potassium channels, GI‐530139. GI‐530139 produces a large maximal enhancement of TREK1 channels with a sub‐micromolar EC_50_. It has improved selectivity for TREK channels over existing compounds such as BL‐1249, and its effect on recombinant hTREK1 channels is seen across several assay platforms including ^86^Rb flux assays and automated and manual patch‐clamp recordings from different expression systems. Within the TREK family, GI‐530139 activates both TREK1 and TREK2 channels but has no effect on TRAAK channels. A concentration of 1 μM is sufficient to substantially enhance current through TREK1 channels across the entire voltage range, including around the resting potential of mammalian neurons such as DRG neurons. Activation of postsynaptic TREK1 channels by a selective activator such as GI‐530159 would hyperpolarize the membrane of DRG neurons and depress neuronal activity in the pain pathway suggesting that such compounds may have potential therapeutic value in the treatment of pain.

From the data in this study using a variety of assay platforms, it is clear that the basal level of activity of TREK1 varies from one assay system to another and, as a consequence, this influences the percentage enhancement of TREK1 current observed. For stably expressed channels in HEK‐293 cells, basal activity of TREK1 channels was very small, so the percentage enhancement seen was large. However, for transiently expressed channels in tsA‐201 cells and stably expressed channels in CHO cells for both automated patch and flux assay systems, basal channel activity is higher, and the percentage enhancements seen are lower but consistent across assays. It is not clear why these differences in basal activity occur between expression systems, but it is important to note that clear enhancement of TREK1 channel activity by GI‐530159 is observed across all the platforms tested. Furthermore, the 50% effective concentration for enhancement of TREK1 channels by GI‐530159 is also consistent across all the platforms tested.

There are a number of compounds that have been identified that activate TREK channels (see Mathie and Veale, 2015; Vivier *et al*., 2016). Fenamate compounds, such as flufenamic acid, are non‐steroidal anti‐inflammatory drugs used clinically in the treatment of pain and are strong activators of TREK channels (Takahira *et al*., 2005; Veale *et al*., 2014) with flufenamic acid the most effective of these compounds (Veale *et al*., 2014). However, none of the fenamates are selective for TREK channels, and all either activate or block many other ion channel types (see Mathie and Veale, 2015, also Guinamard *et al*., 2013).

The dihydroacridine analogue (ML67‐33) has been found to selectively and directly activate TREK1, TREK2 and TRAAK channels (Bagriantsev *et al*., 2013). Bagriantsev *et al*. (2013) showed that ML67‐33 acts *via* a gate located at (or close to) the selectivity filter of the channels, which has been proposed as the site where many different activators converge to regulate channel activity (Schewe *et al*., 2016; Lolicato *et al*., 2017). Enhancement of TREK1 and TREK2 channels is also seen with aristolochic acid, found in a number of plant extracts used to treat pain, and activation by aristolochic acid may occur through a similar mechanism to ML67‐33 (see Veale and Mathie, 2016). However, aristolochic acid is not selective for TREK channels with effects on a range of other K_2P_ channels (Veale and Mathie, 2016).

Another group of compounds that enhance the activity of TREK1 channels is caffeic acid esters [such as cinnamyl 1,3,4‐dihydroxy‐α*‐*cyanocinnamate (CDC) and caffeic acid phenylethyl ester (CAPE)]. It has been suggested that caffeic acid derivatives bind to an external site to produce their effects on TREK1 channels, since activity was retained when the compounds were applied externally in outside‐out patch recordings (Danthi *et al*., 2004). More recently, a range of substituted caffeic acid esters based on a hybrid of CDC and CAPE have been developed, the most promising of which (compound 12U) both enhances the activity of TREK1 channels and displays potent analgesic activity *in vivo* (Rodrigues *et al*., 2014). Very recently, these authors have extended this work by developing a series of substituted acrylic acids, which activate TREK1 channels and show anti‐nociceptive activity *in vivo* (Vivier *et al*., 2017). Also recently, Dadi *et al*. (2017) have shown that PG F2α and a number of other small molecules activate TREK2 channels and stimulate K_2P_ currents in a proportion of DRG neurons. 2‐Aminoethoxydiphenyl borate has also been suggested to be a selective activator of TREK2 channels (Zhuo *et al*., 2015).

Although previous expression studies, including protein expression studies (Maingret *et al*., 2000), suggest that TREK1 channels are expressed in both small‐sized and medium‐sized DRG neurons (Maingret *et al*., 2000; Talley *et al*., 2001; Alloui *et al*., 2006; Dedman *et al*., 2009), both single‐channel and whole‐cell patch recordings suggested that TREK2 channels may also contribute to the background current present in these cells (e.g. Kang and Kim, 2006). Recently, TREK2 channels have been shown to be selectively expressed in IB4 binding, non‐peptidergic C nociceptors (Acosta *et al*., 2014). TREK2 channels contribute to the resting membrane potential of these neurons, since siRNA against TREK2 depolarized the neurons by 10 mV (Acosta *et al*., 2014).

To try to clarify the expression of different TREK channels in different populations of rat DRG neurons, we used single‐cell RNA sequencing. From 120 neurons identified, we found quite variable expression levels of each TREK channel subtype from cell to cell. It is important to note that expression from single‐cell sequencing can be biased to detect highly expressing transcripts, which can lead to biases in classification. The complexity of expression we have observed in rat DRGs is consistent with a recent classification of mouse DRG neurons from transcriptome analysis (Usoskin *et al*., 2015). In that study, unbiased classification of mouse sensory neuron types by large‐scale single‐cell RNA sequencing revealed at least 11 types of sensory neuron, which serves to illustrate the diversity of sensory types and the cellular complexity underlying somatic sensation (Usoskin *et al*., 2015).

Current‐clamp recordings from small DRG neurons in culture, which from our single‐cell RNA sequencing would be likely to express significant TREK1 channels, showed that GI‐530139 significantly hyperpolarized the neurons. This led to a reduction in cell firing frequency; however, this latter effect was variable with frequency reduced or abolished in 57% of cells but unaffected in 43% of cells. From effects on firing, it appears that the neurons fall into two groups, those where firing is blocked and those where there is little effect of GI‐530139, with about half the neurons in each group. This suggests, either, that these small DRG neurons are a heterogeneous group (consistent with the variations seen in TREK channel expression from cell to cell, above) or, alternatively, that the threshold for an effect on firing is slightly different from one group of neurons to another and, at the concentration chosen (1 μM), GI‐530139 only takes a proportion of the neurons below that neuron's particular threshold for firing.

The critical role of TREK channels in pain perception suggests that compounds such as GI‐530139, which selectively enhance their activity, will be of considerable value both experimentally and as potential new analgesic agents targeting these channels (see Mathie and Veale, 2015). As shown in this study, pharmacological activation of TREK channels hyperpolarizes the membrane potential of DRG neurons and depresses neuronal activity. This effect will be primarily through activation of TREK1 channels but, in a subset of neurons, may be through activation of TREK2 channels or even heterodimer TREK1/TREK2 channels (Blin *et al*., 2016; Lengyel *et al*., 2016; Levitz *et al*., 2016). This will act to oppose noxious, excitatory stimulation of these neurons and the subsequent activation of neuronal pain signalling pathways.

## Author contributions

A.J.C.L., A.M., E.B.S., N.C., A.G., L.C., K.O., A.W. and E.L.V. participated in the research design, A.J.C.L., P.P.S., J.L., L.C., B.M.A., S.G.Z., K.Y., E.L.V. and A.W. conducted the experiments, A.J.C.L., P.P.S., J.L., B.M.A., S.G.Z., K.Y., E.L.V., A.W., A.G., A.M., N.C. and E.B.S. performed the data analysis and A.M., E.B.S. and N.C. wrote the manuscript. All authors revised the final version of manuscript and approved its submission.

## Conflict of interest

The authors declare no conflicts of interest.

## Declaration of transparency and scientific rigour

This http://onlinelibrary.wiley.com/doi/10.1111/bph.13405/abstract acknowledges that this paper adheres to the principles for transparent reporting and scientific rigour of preclinical research recommended by funding agencies, publishers and other organisations engaged with supporting research.

## Supporting information


**Figure S1** Activation of hTREK1 current by GI‐530159 in automated patch recordings.
**Figure S2** Activation of hTRAAK current by BL‐1249.
**Figure S3** Single DRG neuron transcriptome – TREK2 channels.
**Figure S4** Single DRG neuron transcriptome – TRAAK channels.
