# Supplementary material for: GI‐530159, a novel, selective, mechanosensitive two‐pore‐domain potassium (K2P) channel opener, reduces rat dorsal root ganglion neuron excitability
Source: Br J Pharmacol. 2017 Dec 29;175(12):2272–83. doi: 10.1111/bph.14098 (PMC5980259; doi:10.1111/bph.14098)
Supplement: Supplementary file 1 — Figure S1 Activation of hTREK1 current by GI‐530159 in automated patch recordings. Figure S2 Activation of hTRAAK current by BL‐1249. Figure S3 Single DRG neuron transcriptome – TREK2 channels. Figure S4 Single DRG neuron transcriptome – TRAAK channels. [file BPH-175-2272-s001.pdf]

Supplementary Information

Supplementary Figure S1: Activation of hTREK1 current by GI-530159 in automated patch recordings.

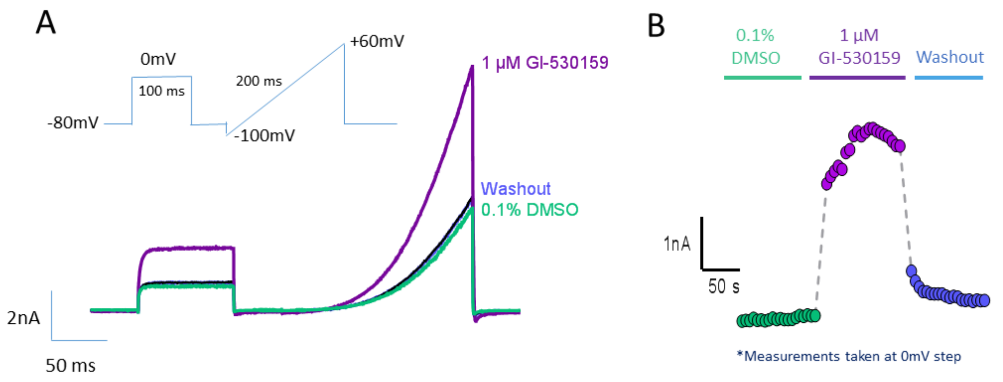

Figure S1: Activation of hTREK1 current by GI-530159 in automated patch recordings.

A) Human TREK1 current elicited by pulse protocol shown recorded on Qpatch 16 automated patch clamp platform after administration of 0.1% DMSO vehicle, 1 µM GI-530159 or after washout with 0.1% DMSO. B) Time course of TREK1 current amplitude recorded at 0 mV on Qpatch platform before during and after washout of 1 µM GI-530159.

Supplementary Figure S2: Activation of hTRAAK current by BL-1249.

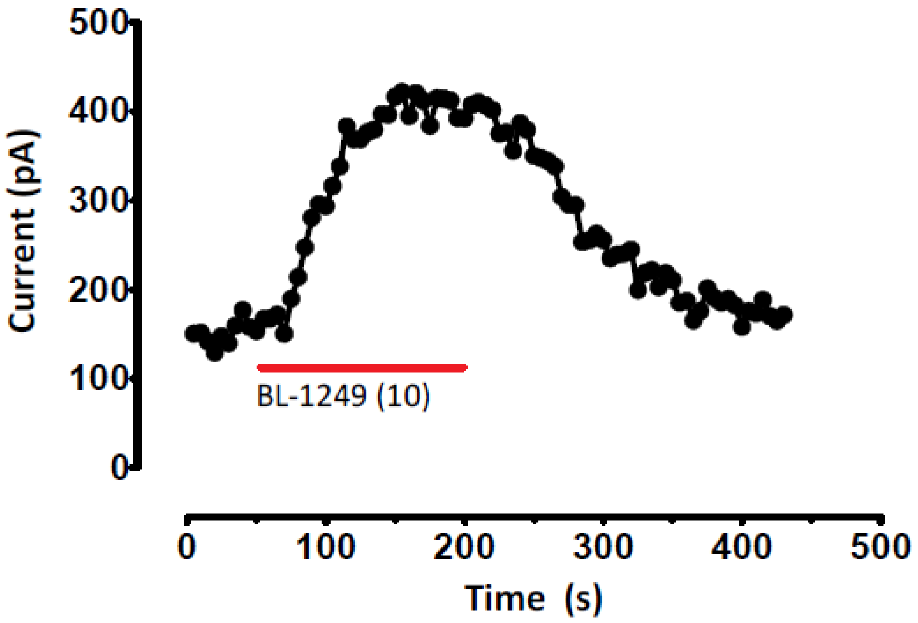

Figure S2: BL-1249 (10 μM) activates hTRAAK channels transiently transfected in tsA-201 cells.

Supplementary Figure S3: Single DRG neuron transcriptome – TREK2 channels.

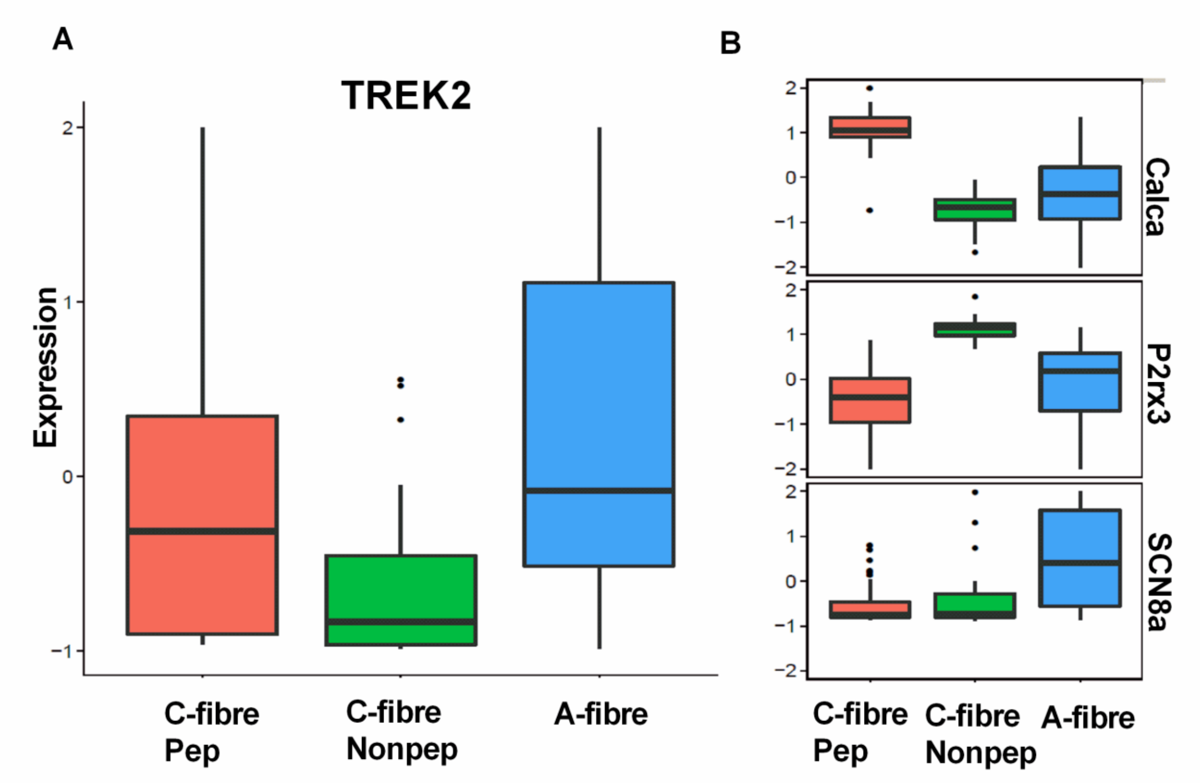

Figure S3. Single DRG neuron transcriptome – TREK2 channels.

A) Differential TREK2 expression in single peptidergic C fibres, Non-peptidergic C fibres and A fibres. B) Comparative expression of selective markers for peptidergic C fibres (Calca), non-peptidergic C fibres (P2rx3) and A fibres (SCN8a). Expression is given as log2 FPKM. Data are from 120 individual DRG neurons isolated from 4 rats.

Supplementary Figure S4: Single DRG neuron transcriptome – TRAAK channels.

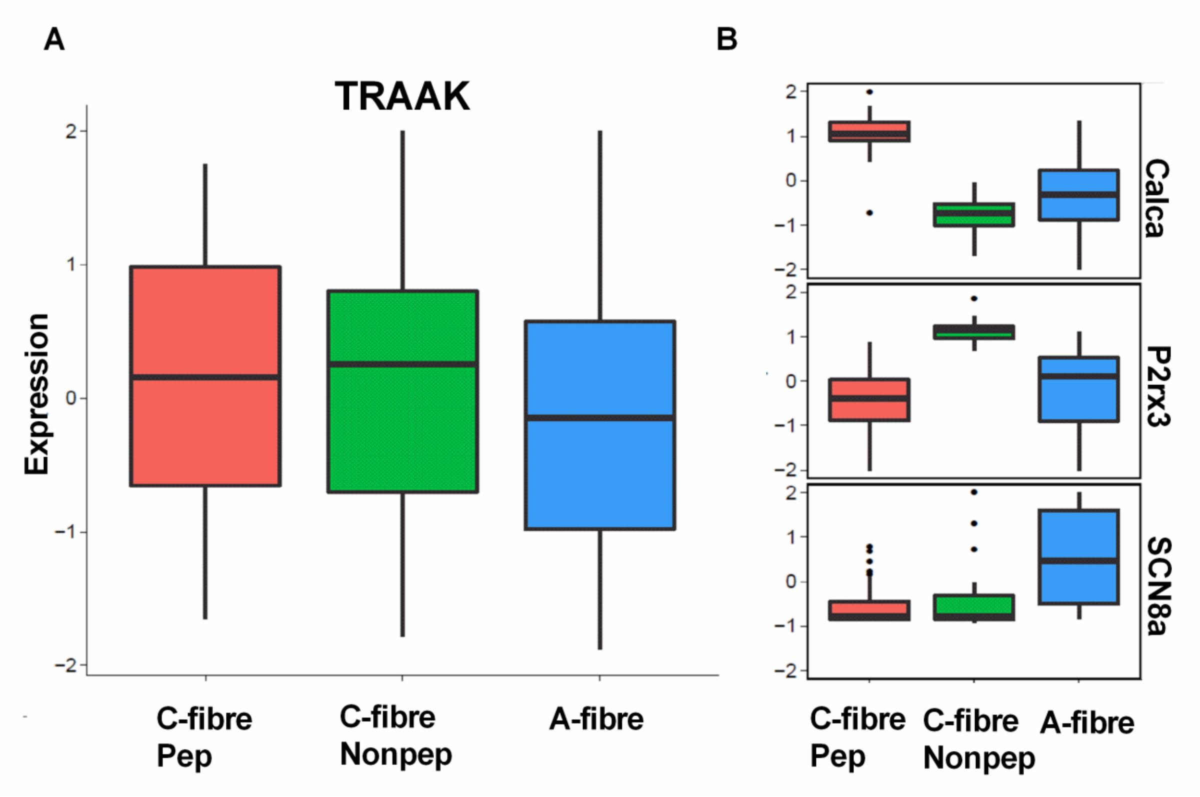

Figure S4. Single DRG neuron transcriptome – TRAAK channels.

A) Differential TRAAK expression in single peptidergic C fibres, Non-peptidergic C fibres and A fibres. B) Comparative expression of selective markers for peptidergic C fibres (Calca), non-peptidergic C fibres (P2rx3) and A fibres (SCN8a). Expression is given as log2 FPKM. Data are from 120 individual DRG neurons isolated from 4 rats.
